# Surgical management of endometriosis: indications, patient preparation, and perioperative planning: Number 6 – 2026

**DOI:** 10.61622/rbgo/2026FPS6

**Published:** 2026-05-20

**Authors:** Agnaldo Lopes da Silva, Sérgio Podgaec, Ricardo de Almeida Quintarios, Júlio César Rosa e Silva

**Affiliations:** Universidade Federal de Minas Gerais Belo Horizonte MG Brazil Universidade Federal de Minas Gerais, Belo Horizonte, MG, Brazil; Universidade de São Paulo São Paulo SP Brazil Universidade de São Paulo, São Paulo, SP, Brazil; Hospital Israelita Albert Einstein São Paulo SP Brazil Hospital Israelita Albert Einstein, São Paulo, SP, Brazil; Universidade do Estado do Pará Belém PA Brazil Universidade do Estado do Pará, Belém, PA, Brazil; Universidade de São Paulo Faculdade de Medicina Ribeirão Preto SP Brazil Faculdade de Medicina, Universidade de São Paulo, Ribeirão Preto, SP, Brazil

## Key points

- Endometriosis is a chronic, heterogeneous disease in which surgical treatment should be selective and individualized.
- Symptom burden, disease phenotype, impact on quality of life, and reproductive goals are more relevant than disease stage in guiding surgical decision making.
- Surgery is not first-line therapy and offers limited benefit in women with mild symptoms or adequate response to medical treatment.
- Clear surgical benefit is more consistently observed in symptomatic ovarian endometriomas and deep infiltrating endometriosis associated with organ dysfunction.
- Preoperative assessment is critical to balance expected benefit against surgical risk, particularly regarding ovarian reserve and long-term morbidity.
- Surgery is not curative and should be framed as one component of a longitudinal management strategy, frequently requiring postoperative medical therapy.
- Complex disease phenotypes and fertility-preserving surgery benefit from specialized or multidisciplinary care.

## Recommendations

- Surgical treatment should be considered only in women with clinically significant symptoms, organ dysfunction, or well-defined clinical indications.
- Women with mild symptoms or adequate control with medical therapy should be managed conservatively.
- Preoperative evaluation should include structured transvaginal ultrasound and pelvic MRI when deep disease is suspected, to guide surgical planning and counseling.
- Assessment of ovarian reserve should be considered before surgery in women with ovarian endometriomas and reproductive plans, particularly in cases of bilateral or repeat surgery.
- Patients should be counseled preoperatively regarding realistic expectations, recurrence risk, potential impact on fertility, and the role of long-term postoperative management.
- Structured perioperative care pathways, including Enhanced Recovery After Surgery (ERAS) protocols, should be implemented in endometriosis surgery to optimize postoperative recovery, reduce morbidity, and improve patient experience.
- Referral to specialized or multidisciplinary centers should be considered for deep infiltrating disease, complex ovarian endometriomas, or fertility-preserving surgery with anticipated high complexity.

## Background

Endometriosis is a chronic and heterogeneous disease associated with pelvic pain, infertility, and impaired quality of life. Surgical treatment is not first-line therapy and should be reserved for selected clinical scenarios in which medical management is ineffective, contraindicated, or inappropriate. Clear indications include persistent or severe pain refractory to optimized hormonal therapy, intolerance or contraindication to medical treatment, ovarian endometriomas with specific indications such as large size or suspicious imaging features, deep infiltrating disease associated with organ dysfunction, and selected cases of endometriosis-associated infertility in which anatomical correction may improve outcomes or when assisted reproduction is not preferred or feasible.^(1–4)^

Disease phenotype and symptom burden are central to surgical decision making, as benefits and risks differ substantially among superficial peritoneal disease, ovarian endometriomas, and deep infiltrating endometriosis.^(2–5)^ Patient priorities, particularly reproductive goals and impact on quality of life, must be explicitly integrated into the indication for surgery.^(3–6)^

Preoperative assessment should include comprehensive clinical evaluation, structured imaging with transvaginal ultrasound and/or magnetic resonance imaging for disease mapping, and fertility assessment when relevant.^(2,4,6)^ In complex cases, particularly deep infiltrating disease, multidisciplinary planning is recommended to optimize outcomes and minimize complications.^(1,3,5)^

Because surgery is not curative and recurrence is common, counseling should address realistic expectations, potential impact on ovarian reserve, perioperative risks, and the likely need for postoperative medical therapy when pregnancy is not immediately desired.^(1,2,4)^

**Figure 1:**
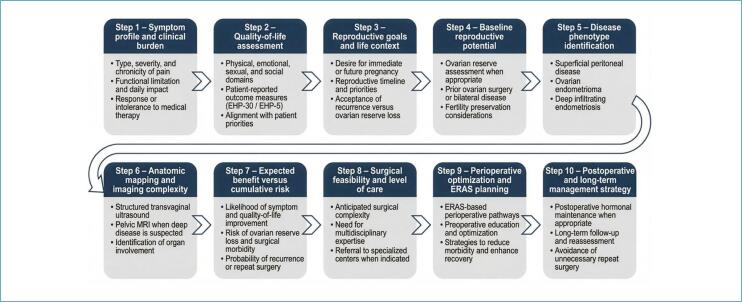
Preoperative decision-making framework for surgical management of endometriosis

This Position Statement defines the appropriate indications for surgical treatment of endometriosis and emphasizes selective, individualized decision making based on disease phenotype, symptom severity, quality of life, and reproductive goals. Figure 1 summarizes a structured, multidimensional preoperative framework designed to guide appropriate surgical indication and planning in women with endometriosis.

## What are the clear clinical indications for surgical treatment in women with endometriosis?

**Figure 2:**
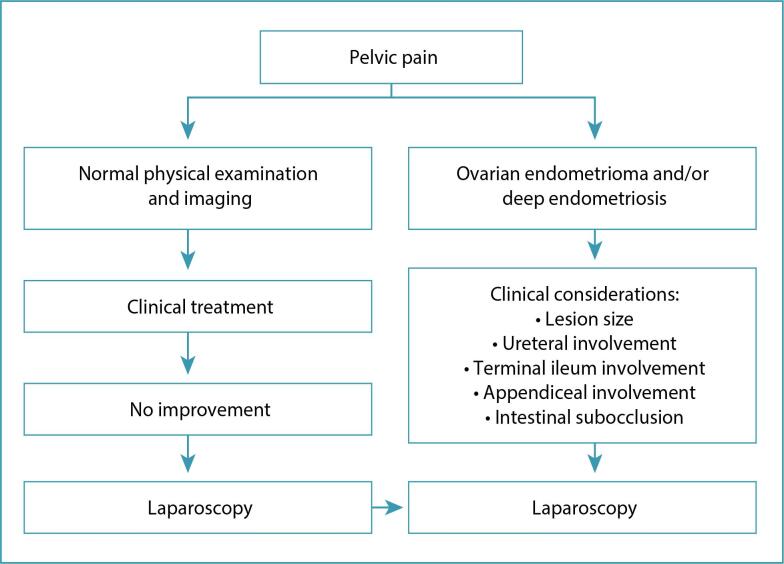
Therapeutic flowchart for patients with clinical suspicion of pelvic pain associated with endometriosis (FEBRASGO 2023)

Surgical treatment has a defined role in the management of endometriosis and should be reserved for clearly established clinical scenarios, particularly when medical therapy is ineffective, not tolerated, or contraindicated.^(2,4)^Figure 2 presents a FEBRASGO-based therapeutic flowchart integrating clinical presentation, imaging findings, and treatment response to support selective surgical decision making in endometriosis-related pelvic pain.^(7)^

Ovarian endometriomas represent an important indication for surgery when specific clinical or imaging criteria are present. Surgical treatment may be considered in cases of persistent symptoms despite optimized medical therapy, imaging features suspicious for malignancy, or when endometriomas contribute to infertility, pelvic distortion, or interfere with assisted reproductive techniques. In these scenarios, surgery may be required for symptom control, diagnostic clarification, or individualized fertility-oriented management.^(2,8)^

Surgery is clearly indicated in cases of deep infiltrating endometriosis associated with organ dysfunction. Women presenting with urinary or gastrointestinal involvement, including hematuria, hematochezia, or obstructive symptoms affecting the bowel or urinary tract, particularly when there is evidence of organ compromise, may benefit from surgical treatment performed in specialized centers with multidisciplinary expertise.^(2)^

Endometriosis-associated infertility represents a more nuanced indication. Surgical treatment may be considered in selected women, particularly those with mild to moderate disease or when endometriomas, adhesions, or pelvic distortion are likely to impair tubal function or interfere with oocyte retrieval. In these cases, potential reproductive benefits must be carefully weighed against the risk of reduced ovarian reserve, especially in women with ovarian endometriomas.^(1,8,9)^

Surgery is also indicated in situations of acute presentation or diagnostic uncertainty, such as acute pelvic pain, adnexal masses with concern for malignancy, or scenarios requiring immediate diagnosis and treatment.^(4,5)^

In women with persistent, severe symptoms refractory to conservative management and who no longer desire future fertility, definitive surgical treatment may be considered. Hysterectomy with complete excision of visible disease may be an option in carefully selected patients with refractory pain, particularly when associated with other uterine disorders such as adenomyosis and leiomyomatosis.^(2)^

Surgical decision making in endometriosis should be individualized and based on symptom severity, disease phenotype, reproductive goals, and patient preferences. Potential risks, including surgical complications and adverse effects on ovarian reserve, must be balanced against expected benefits, highlighting the importance of shared decision making and referral to experienced centers for complex procedures.^(2,3,8)^

## Which patients with endometriosis are unlikely to benefit from surgery and should be managed conservatively?

Women with mild or asymptomatic endometriosis, as well as those whose symptoms are adequately controlled with first-line hormonal therapies, are unlikely to benefit from surgical treatment and should generally be managed conservatively. In this population, surgery does not provide additional benefit in terms of pain control or quality of life and exposes patients to unnecessary operative risks.^(2–4,10)^

Women who do not desire future fertility and who do not present severe pain, organ dysfunction, or large ovarian endometriomas can usually be managed effectively with long-term suppressive medical therapy. Continuous hormonal regimens, including combined oral contraceptives, progestin-only therapies, and intrauterine systems, provide sustained symptom relief and are preferred in the absence of clear surgical indications.^(1,4,6)^

Conservative management is particularly appropriate for women with contraindications to surgery, increased surgical risk, or significant comorbidities, in whom the potential benefits of surgery are outweighed by the risks of perioperative complications, symptom recurrence, and cumulative harm, especially after repeated or extensive procedures that may further compromise ovarian reserve.^(1,6,11)^

In women with centralized pain phenotypes, multisite pain, or persistent symptoms after prior surgery, further operative intervention rarely provides sustained benefit and may exacerbate pain chronicity. In these cases, treatment should shift toward a multimodal conservative strategy, including neuromodulation and other approaches targeting central pain mechanisms.^(1,6,11,12)^

Current consensus supports a selective, symptom-focused approach, reserving surgery for women with refractory symptoms, organ dysfunction, or infertility not amenable to medical therapy. For the majority of women without these indications, long-term hormonal suppression and multidisciplinary pain management, including pelvic floor physiotherapy for assessment and treatment of associated myofascial dysfunction, remain the cornerstone of treatment.^(1,4,6,11,13,14)^

## How should symptom burden, disease phenotype, and impact on quality of life guide the indication for surgery?

Symptom burden is the primary determinant for recommending surgical treatment in women with endometriosis. Surgery should be considered mainly in women with severe, persistent, or hormone-resistant pain that substantially impairs daily activities or quality of life, particularly when medical therapy is ineffective, contraindicated, or not tolerated.^(2–4,6)^ In contrast, women with mild symptoms, incidental disease, or symptoms adequately controlled with hormonal therapy are unlikely to derive additional benefit from surgery and should generally be managed conservatively.^(2–4,6)^

Disease phenotype significantly influences the expected benefit from surgery. In superficial peritoneal endometriosis, evidence supporting surgical intervention is limited, and pain relief is often inconsistent, favoring conservative management unless symptoms are clearly refractory.^(2,15)^ Ovarian endometriomas warrant surgical consideration primarily when they are large, symptomatic, suspicious on imaging, or interfere with fertility treatment. However, excisional surgery is consistently associated with a reduction in ovarian reserve, requiring careful counseling in women desiring future fertility.^(3,8,16)^

Deep infiltrating endometriosis involving the bowel, bladder, or ureter and associated with organ dysfunction represents a clear indication for surgical treatment. In these cases, surgery may relieve symptoms, prevent progressive organ damage, and improve quality of life, and should be performed by experienced multidisciplinary teams in specialized centers.^(2,6)^

Quality of life impact and patient-centered decision making are central to treatment selection. Assessment should incorporate not only pain intensity but also functional limitation, psychological distress, and alignment with patient goals and preferences. Women with predominantly centralized or multisite pain are less likely to benefit from surgery and should preferentially be managed with multimodal conservative strategies.^(2,4,6,14)^

Surgical treatment should be reserved for women with substantial symptom burden, phenotype-specific indications, or significant impact on quality of life, integrating reproductive goals and patient preferences within a shared decision-making framewor.^(2–4,6,8,14–17)^

## Should surgical decision making be based on disease stage or on an individualized clinical context?

Surgical decision making in endometriosis should be guided by an individualized clinical context, integrating symptom burden, disease phenotype, impact on quality of life, and reproductive goals, rather than being based primarily on disease stage. The revised American Society for Reproductive Medicine (rASRM) classification has limited ability to predict pain severity, response to treatment, or prognosis. Women with minimal disease may experience severe symptoms, whereas others with advanced disease may remain asymptomatic, underscoring the poor correlation between anatomic staging and clinical presentation.^(3,18)^

Contemporary guidelines and reviews consistently discourage the use of disease stage alone to guide surgical indications. Surgery is primarily recommended for women with severe or refractory symptoms, organ dysfunction, or infertility not amenable to medical therapy, while routine surgical intervention is not indicated in asymptomatic women or in those with mild disease well controlled with medical treatment.^(2,6,16)^

Disease phenotype provides more clinically relevant information than stage. Deep infiltrating endometriosis, large or suspicious ovarian endometriomas, and lesions associated with bowel, bladder, or ureteral involvement are more likely to benefit from surgical intervention. In parallel, patient priorities—including fertility goals, the potential risk of ovarian reserve loss, and the psychosocial impact of symptoms—must be central to shared decision making and transparent counseling regarding potential benefits and risks.^(2–6,19,20)^

Despite advances in surgical techniques, important evidence gaps persist. High-quality data linking specific phenotypes and symptom profiles to optimal surgical outcomes remain limited, and current classification systems do not adequately capture disease complexity or predict long-term benefit. There is a clear need for improved phenotypic stratification and prospective studies focused on patient-centered outcomes.^(14,17,18,21)^

Current consensus supports a selective, symptom-driven approach to surgery in endometriosis. Surgical intervention should be reserved for women with significant symptom burden, disease phenotypes associated with organ compromise or reproductive impairment, or substantial impact on quality of life, and should not be determined solely by disease stage.^(2,3,6,14,16–22)^

## What are the realistic and predefined objectives of endometriosis surgery, and how should they influence surgical planning and extent?

The objectives of surgical treatment for endometriosis must be realistic, predefined, and aligned with patient-centered outcomes. Surgery aims to reduce pain, restore pelvic anatomy, manage organ dysfunction, improve fertility in selected cases, and exclude malignancy. Importantly, surgery is not curative, and recurrence remains common; postoperative medical therapy is frequently required to reduce recurrence risk and support long-term disease control.^(1–4,10,21)^

Surgical planning and extent should be guided by symptom burden, disease phenotype, and impact on quality of life, rather than disease stage alone. Symptom intensity and functional impairment represent the primary determinants for surgical indication, as disease stage poorly predicts pain severity or treatment response.^(1–4)^

Accordingly, surgery should be considered mainly for women with severe, persistent, or hormone-resistant pain, for those with large or suspicious ovarian endometriomas, for deep infiltrating disease associated with bowel, bladder, or ureteral dysfunction, and for selected cases of infertility not amenable to assisted reproductive technologies. Importantly, surgical intervention may also be indicated in selected asymptomatic patients when endometriotic disease poses a risk of progressive organ damage, such as terminal ileum involvement, appendiceal or cecal disease, ureteral lesions associated with hydronephrosis, or deep rectal involvement affecting more than 50% of the bowel circumference. In these scenarios, the indication for surgery is driven by the risk of functional compromise rather than symptom burden. Conversely, women with mild symptoms, incidental disease, or symptoms adequately controlled with medical therapy are unlikely to benefit from surgery and should generally be managed conservatively.^(1–5,16,19)^

Disease phenotype has direct implications for surgical strategy. Superficial peritoneal disease does not consistently benefit from extensive excision, whereas ovarian endometriomas and deep infiltrating endometriosis often require more complex procedures, ideally performed in experienced multidisciplinary centers. In these scenarios, potential benefits must be carefully balanced against well-recognized risks, including loss of ovarian reserve, early and late postoperative complications, and disease recurrence, particularly in women desiring future fertility.^(1–5,16,19)^

Shared decision making and interdisciplinary care are essential. Surgical objectives and extent should be explicitly aligned with patient priorities, reproductive goals, and quality-of-life expectations, using patient-reported outcome measures whenever possible. Conservative and fertility-sparing strategies are preferred when appropriate, while more extensive procedures may be required in the presence of organ dysfunction or malignancy risk.^(5,14,16,19,21)^

The objectives of endometriosis surgery—pain reduction, functional improvement, fertility optimization in selected cases, management of organ involvement, and exclusion of malignancy—should directly inform the indication, timing, and extent of surgery. Surgical decision making must remain individualized, symptom-driven, and patient-centered, with careful balancing of benefits against the risks of recurrence, ovarian reserve loss, and surgical morbidity.^(1–5,10,14,16,19,21)^

## How should reproductive goals influence the indication, timing, and extent of surgical treatment?

Reproductive goals should play a central role in defining the indication, timing, and extent of surgical treatment in women with endometriosis. Given the chronic, estrogen-dependent, and heterogeneous nature of the disease, management must be individualized and patient-centered, particularly when fertility preservation or conception is a priority.^(1–3,10,21,23)^

In women desiring immediate or future fertility, decisions regarding surgery, medical management, expectant treatment, or assisted reproductive technologies (ART) should be based on a careful balance between potential benefits and risks. Surgical treatment may improve spontaneous conception in selected women, particularly those with mild to moderate disease; however, it should not be routinely recommended for all infertile patients and is not a substitute for ART.^(6,16,19–21)^

ART is generally preferred as first-line therapy for endometriosis-associated infertility, especially in the presence of additional infertility factors, reduced ovarian reserve, or when rapid conception is desired. Surgery may be considered in selected cases to restore pelvic anatomy, improve natural fertility, facilitate access to the ovaries for ART or in the presence of hydrosalpinx, where salpingectomy provides a clear benefit prior to ART, but requires careful counseling regarding expected benefit, potential delay in conception, and the risk of ovarian reserve loss.^(6,16,19,21)^

Timing of surgery should reflect symptom burden, disease phenotype, and fertility priorities. Although early intervention may benefit women seeking spontaneous conception, the potential negative impact on ovarian reserve, particularly after excision of ovarian endometriomas, must be explicitly discussed. Preoperative fertility assessment and, when appropriate, multidisciplinary evaluation are recommended. In women with severe pain, organ dysfunction, or large or suspicious endometriomas, surgery may be indicated regardless of reproductive plans, but timing should be coordinated with fertility strategies whenever possible.^(3,8,16,19,21)^

The extent of surgery should be tailored to disease phenotype and reproductive intent. Reproductive-sparing techniques and conservative excision strategies are preferred in women prioritizing fertility. More radical procedures, including hysterectomy or extensive excision, should be reserved for refractory disease or for women who do not desire future fertility, given their long-term implications for ovarian function and overall health.^(3–5,8,16,19,21)^

Reproductive goals should directly influence the indication, timing, and extent of surgical treatment in endometriosis. Management should integrate symptom burden, disease phenotype, and quality-of-life impact within an individualized, multidisciplinary, and fertility-preserving framework. Shared decision making is essential to balance the potential benefits of surgery against the risks of ovarian reserve loss, recurrence, and delayed conception.^(1–6,8,10,16,19–21)^

## What preoperative clinical, imaging, and laboratory evaluation is essential before endometriosis surgery?

A comprehensive, patient-centered preoperative assessment is essential before surgical treatment of endometriosis. Evaluation should integrate symptom burden, disease phenotype, impact on quality of life, and reproductive goals to guide surgical indication, timing, and extent, emphasizing individualized decision making rather than disease stage alone.^(1,2,5,19,21)^

Clinical assessment should include a detailed history focusing on pain characteristics, response to prior treatments, menstrual and fertility history, previous surgeries, and the functional and psychosocial impact of symptoms. Physical examination should include speculum and bimanual evaluation, with attention to pelvic tenderness, uterosacral nodularity, adnexal masses, reduced uterine mobility, and signs suggestive of deep infiltrating disease.^(2,4,24)^

Imaging plays a central role in preoperative planning. Transvaginal ultrasound is recommended as the first-line imaging modality for suspected endometriosis, particularly for the detection of ovarian endometriomas and deep pelvic disease, especially when performed by experienced operators using structured protocols and, when appropriate, with bowel preparation to improve visualization of posterior compartment lesions, in patients desiring future fertility, an antral follicle count (AFC) should be performed. Pelvic magnetic resonance imaging is advised when deep infiltrating disease is suspected, especially with possible bowel, bladder, or ureteral involvement, or when detailed mapping is required to anticipate surgical complexity and multidisciplinary needs.^(4,21,24,25)^

Laboratory testing has limited diagnostic value. Serum CA-125 is not recommended for routine assessment due to low specificity. In women with ovarian endometriomas and fertility concerns, evaluation of ovarian reserve, particularly anti-Müllerian hormone levels, is recommended to inform counseling regarding surgical risks and fertility preservation strategies.^(3,19,21)^

Formal assessment of quality of life and reproductive goals using validated patient-reported outcome measures is encouraged. Clarification of fertility intentions should directly influence surgical planning, with reproductive-sparing approaches prioritized when future fertility is desired.^(3,5,19,21)^

Multidisciplinary planning is recommended for suspected or confirmed deep infiltrating disease or complex presentations. Referral to specialized centers and coordination with colorectal surgeons, urologists, fertility specialists, and pain teams should be considered to reduce perioperative risk and optimize outcomes.^(1,19,21,24)^

Essential preoperative evaluation before endometriosis surgery includes detailed clinical assessment, structured transvaginal ultrasound and pelvic MRI for disease mapping, selective laboratory evaluation including ovarian reserve when appropriate, formal assessment of quality of life and reproductive goals, and multidisciplinary planning for complex disease. These elements should directly inform surgical indication, timing, and extent.^(1–5,19,21,24,25)^

## How should advanced imaging and preoperative disease mapping influence surgical strategy and patient counseling?

Advanced imaging and preoperative disease mapping should directly inform surgical strategy and patient counseling in endometriosis by allowing accurate characterization of disease phenotype, extent, and anatomic distortion. Imaging-guided assessment supports individualized decision making based on symptom burden, impact on quality of life, and reproductive goals, and reflects the transition from a surgically defined diagnosis to a clinically and imaging-based approach.^(2,4,21,26)^

Transvaginal ultrasound is the recommended first-line imaging modality because of its accessibility and cost effectiveness, with high diagnostic accuracy for ovarian endometriomas and moderate sensitivity for deep infiltrating disease. When performed using structured protocols by experienced operators, advanced ultrasound techniques, including dynamic assessment and compartment-based evaluation, improve detection of adhesions and deep lesions and provide relevant information for surgical planning.^(2,21,27–30)^

Magnetic resonance imaging offers superior sensitivity for deep infiltrating disease and allows panoramic pelvic and extrapelvic assessment with lower operator dependency. It is particularly valuable for preoperative mapping of bowel, bladder, and ureteral involvement, evaluation of complex anatomic distortion, and assessment of malignancy risk. However, both ultrasound and MRI have limited sensitivity for superficial peritoneal disease, and negative imaging does not exclude endometriosis.^(21,26–29,31,32)^

Structured preoperative disease mapping using compartment-based and pattern-recognition approaches facilitates surgical planning, interdisciplinary communication, and appropriate referral. Ultrasound is particularly useful for dynamic evaluation of adhesions and bowel wall infiltration, whereas MRI provides a comprehensive overview of disease distribution and spatial relationships, supporting multidisciplinary surgical planning when needed.^(21,25,28–32)^

Imaging findings should guide the choice of surgical approach, extent of resection, and need for multidisciplinary involvement. Extensive deep infiltrating disease affecting the bowel, bladder, or ureter may require complex surgery in specialized centers, while isolated ovarian endometriomas may be managed with fertility-sparing cystectomy. Imaging also helps identify patients at increased risk of surgical complications or ovarian reserve impairment, supporting individualized counseling and risk stratification.^(2,6,21,28,31)^

Patient counseling should incorporate imaging findings to establish realistic expectations regarding surgical complexity, likelihood of complete excision, potential need for staged or multidisciplinary procedures, and anticipated impact on pain, fertility, and recurrence. Imaging-based counseling strengthens shared decision making by aligning surgical objectives with patient priorities and quality-of-life considerations.^(6,17,21,26,27)^

Despite advances, limitations persist, including variability in imaging expertise, lack of universal standardization of protocols, and unequal access to advanced imaging. Emerging tools, including artificial intelligence-assisted interpretation and multimodal imaging strategies, may further improve diagnostic accuracy and individualized care but require validation before routine implementation.^(21,26,27,29)^

Advanced imaging and preoperative disease mapping are integral to individualized surgical strategy and patient counseling in endometriosis. Imaging-guided assessment enables tailored surgical planning, supports multidisciplinary care, and underpins informed shared decision making aligned with contemporary recommendations.^(2,4,6,17,21,25–32)^

## What is the role of intentional preoperative hormonal suppression before endometriosis surgery?

Preoperative hormonal treatment aims to suppress ovarian activity, reduce inflammation, and induce regression of endometriotic lesions, potentially improving symptom control and facilitating surgical planning. This rationale is grounded in the estrogen-dependent and inflammatory pathophysiology of endometriosis; however, its routine use before surgery remains controversial.^(2,3,11,21,33,34)^

Available pharmacological options include combined estrogen–progestin therapies, progestins, and gonadotropin-releasing hormone agonists or antagonists. These agents suppress ovulation and menstrual activity, induce atrophy of ectopic endometrial tissue, and reduce inflammatory mediators. While effective for symptom control, their specific impact on surgical outcomes is limited and inconsistent.^(2,3,11,33)^

Evidence from randomized trials and systematic reviews does not support routine preoperative hormonal suppression for all patients. Studies have not consistently demonstrated reductions in postoperative pain, recurrence rates, or improvements in fertility outcomes compared with surgery alone. A major Cochrane review concluded that preoperative medical therapy does not confer clear additional benefit in most cases, a position reflected in contemporary guideline-based recommendation.^(21,33,35,36)^

Preoperative hormonal therapy may be considered in selected clinical scenarios. These include women with severe pain requiring symptom control prior to surgery, extensive or deep infiltrating disease with marked inflammatory activity, large ovarian endometriomas where temporary suppression may assist in preoperative assessment, or patients requiring optimization before surgery due to comorbidities or increased operative risk. In fertility-preserving strategies, hormonal suppression may also be used temporarily to allow oocyte or embryo cryopreservation before surgical excision of endometriomas.^(2,4,10,11,21,36)^

Preoperative hormonal therapy is not recommended in women with immediate fertility goals, as ovulation suppression delays conception, nor in patients who are nonresponders to medical therapy or have contraindications to hormonal treatment. Its use should be individualized, guided by symptom burden, disease phenotype, quality-of-life impact, and reproductive planning, with clear counseling regarding expected benefits and limitations.^(4,21,33,36,37)^

Significant evidence gaps remain regarding optimal agent selection, duration of therapy, and identification of patient subgroups most likely to benefit. Further high-quality studies are needed to better integrate preoperative hormonal strategies into personalized surgical care pathways.^(33,35–37)^

Preoperative hormonal treatment may be considered in selected women with severe symptoms, extensive or deep disease, or specific clinical needs before surgery, but it is not routinely recommended. Decisions should be individualized and aligned with symptom burden, disease phenotype, quality of life, and reproductive goals, recognizing the limited and heterogeneous evidence base.^(2–4,10,11,21,33–37)^

## How should patients be counseled preoperatively regarding expected benefits, risks, recurrence, and alternative treatment options?

Preoperative counseling for endometriosis surgery should be patient-centered and structured around shared decision making, integrating symptom burden, disease phenotype, impact on quality of life, and reproductive goals. Counseling must emphasize that surgery is not curative and that outcomes vary according to disease characteristics and individual patient factors.^(1,16,20,21)^

Expected benefits of surgery include pain reduction, improvement in quality of life, restoration of pelvic anatomy, and potential enhancement of fertility in selected cases. Surgical treatment is most effective for deep infiltrating disease, large or symptomatic ovarian endometriomas, hormone-resistant pain, and cases associated with organ dysfunction. Benefits are less predictable for superficial peritoneal disease, and patients should be counseled accordingly.^(1–5,9,20,38)^

Risks must be discussed transparently and include bleeding, infection, injury to pelvic organs, and loss of ovarian reserve, particularly after excision of ovarian endometriomas. Recurrence is common; longitudinal studies show pain recurrence rates of approximately 40–45%, with 15–20% of women requiring repeat surgery within two years and up to 50% undergoing reoperation within five to seven years. Persistent or recurrent pain is more frequent in women with centralized or multifactorial pain syndromes.^(1–5,38)^

Postoperative recurrence risk and long-term management should be addressed preoperatively. For women not seeking immediate pregnancy, postoperative hormonal suppression with combined oral contraceptives, progestins, or levonorgestrel-releasing intrauterine systems is recommended to reduce recurrence and maintain symptom control. Long-term medical therapy after surgery is required for most patients.^(2,10,21,35,37)^

Alternative treatment options should be clearly presented, including nonsteroidal anti-inflammatory drugs, hormonal therapies, and expectant management. Surgery should generally be reserved for women in whom medical therapy is ineffective, contraindicated, or not tolerated, or for those with deep disease or large endometriomas causing significant symptoms or organ compromise. In infertility, counseling must emphasize that surgery may improve spontaneous conception in selected cases but does not replace assisted reproductive technologies, which remain the most effective strategy for many patients.^(2,4,5,10,20,21)^

Patients should also be informed about existing uncertainties, including limited high-quality comparative data on long-term pain relief, fertility outcomes, and quality-of-life benefits across disease phenotypes and surgical techniques. These uncertainties should be incorporated into counseling to support realistic expectations and informed consent.^(2,9,21,38)^

Preoperative counseling should clearly communicate that surgery may improve symptoms and fertility in selected women but carries relevant risks, including recurrence and potential ovarian reserve loss. Long-term medical therapy is frequently required, and alternative medical and reproductive options must be discussed. Surgical decisions should be individualized and aligned with symptom severity, disease phenotype, quality of life, and reproductive goals.^(1–5,9,10,16,20,21,35,37,38)^

## What is the role of structured perioperative care pathways, including Enhanced Recovery After Surgery (ERAS) programs, in endometriosis surgery?

Structured perioperative care pathways, including Enhanced Recovery After Surgery programs, play a central role in contemporary endometriosis surgery by reducing postoperative pain, accelerating recovery, and improving patient satisfaction across different disease phenotypes, including deep infiltrating and ovarian endometriosis.^(39–49)^

Key ERAS components include multimodal opioid-sparing analgesia, early mobilization, early enteral feeding, shortened fasting with carbohydrate loading, avoidance of routine mechanical bowel preparation, and structured preoperative education. These measures are associated with significant reductions in opioid consumption, improved early pain control, faster gastrointestinal recovery, and shorter hospital stay.^(40–43,47)^

Multimodal analgesia strategies reduce perioperative opioid use by up to 60%, with lower early postoperative pain scores. Early mobilization and feeding decrease postoperative ileus and shorten time to gastrointestinal recovery, while preoperative carbohydrate loading attenuates the surgical stress response and improves perioperative metabolic stability.^(40–43)^

Structured patient education and expectation management are integral to recovery pathways and are associated with higher patient satisfaction, improved functional recovery, and increased rates of same-day or early discharge without higher complication or readmission rates. Preoperative counseling within ERAS frameworks should be viewed as a therapeutic intervention rather than a purely informational step.^(40,44,45,48)^

In women undergoing surgery for deep infiltrating endometriosis, ERAS implementation reduces length of stay by approximately one to one and a half days and is associated with lower postoperative morbidity, even in complex procedures such as bowel resection. These protocols do not negatively affect fertility outcomes. Preservation of ovarian reserve depends primarily on surgical technique rather than perioperative pathways, although faster recovery may indirectly facilitate reproductive planning.^(8,39,48,50)^

ERAS protocols should be adapted to disease complexity, symptom burden, and patient-specific factors, including chronic pelvic pain, centralized pain syndromes, and prior opioid use. Their impact on fertility is indirect and mediated through reduced morbidity and faster recovery rather than direct reproductive effects.^(8,50,51)^

Structured perioperative care pathways, including ERAS programs, are an essential component of high-quality surgical management in endometriosis. When appropriately tailored and consistently applied, they improve recovery, reduce postoperative pain and opioid use, shorten hospitalization, and enhance patient-centered care without compromising reproductive outcomes. Implementation of ERAS pathways should be considered a quality benchmark in endometriosis surgery.

## When should endometriosis surgery be referred to specialized or multidisciplinary centers?

Endometriosis surgery should be referred to specialized or multidisciplinary centers when patients present with deep infiltrating disease, large or complex ovarian endometriomas, involvement of bowel, ureter, bladder, or thoracic structures, severe or refractory symptoms, organ dysfunction, or when fertility preservation is a priority in the context of anticipated high surgical complexity.^(1–4,10,16,17,19,21,52)^

Referral is particularly indicated when first-line medical therapies are ineffective, not tolerated, or contraindicated, and in the presence of clinically relevant organ compromise, such as hydronephrosis, bowel obstruction, hematuria, or hematochezia, or when there is suspicion of malignancy. Contemporary international recommendations emphasize that referral decisions should be individualized and based on symptom burden, disease phenotype, imaging findings, and reproductive goals, within a shared decision-making framework.^(1,2,4,10,16,17,19)^

Deep infiltrating endometriosis frequently involves complex anatomic sites, including the rectosigmoid colon, bladder, ureters, and, less commonly, thoracic structures. Large or atypical ovarian endometriomas, particularly those exceeding five centimeters, further increase surgical complexity and risk. These scenarios require advanced imaging, structured preoperative disease mapping, and high-level surgical expertise, as procedures may involve ureterolysis, bowel resection, or coordinated thoracic intervention, with potential implications for fertility and postoperative morbidity.^(1–3,17,52)^

Evidence from observational cohorts and guideline-based recommendations supports management of complex endometriosis in high-volume centers with coordinated multidisciplinary teams. These teams typically include gynecologic surgeons with advanced minimally invasive expertise, colorectal surgeons, urologists, radiologists, anesthesiologists, and reproductive specialists when appropriate. Such an approach is associated with improved surgical completeness, lower complication rates, and better functional outcomes, particularly in patients with deep infiltrating disease and multi-organ involvement.^(1,3,17,21,52)^

Disease phenotype and symptom severity should guide referral decisions. Deep infiltrating disease and large endometriomas are associated with longer operative times, higher complication risk, and increased likelihood of ovarian reserve impairment, reinforcing the importance of experienced teams and multidisciplinary planning. Fertility preservation considerations, including the risk of diminished ovarian reserve after endometrioma excision, should be explicitly discussed and incorporated into referral pathways and surgical strategy.^(3,16,19,21)^

Quality-of-life impact and reproductive goals must be systematically assessed and integrated into surgical planning. Use of validated patient-reported outcome measures is encouraged to quantify symptom burden and support individualized care. Preoperative counseling should address expected benefits, recurrence risk, the potential need for postoperative medical therapy, and long-term implications for fertility, particularly in reproductive-age women undergoing complex ovarian or deep pelvic surgery.^(1–3,16,17)^

Referral to specialized or multidisciplinary centers is recommended for women with deep infiltrating endometriosis, large or complex endometriomas, organ involvement, severe or refractory symptoms, or when fertility preservation is a priority in the context of high surgical complexity. This approach ensures access to advanced imaging, coordinated interdisciplinary care, and expertise in complex surgical techniques, optimizing outcomes while minimizing risks, in accordance with current international guideline-based recommendations and best clinical practice.^(1–4,10,16,17,19,21,52)^ Certain clinical scenarios require referral to specialized or multidisciplinary centers in order to optimize outcomes, minimize morbidity, and appropriately address fertility preservation and organ-specific risks (Table 1).

**Table 1:** Indications for referral to specialized or multidisciplinary centers

Clinical scenario	Rationale for referral	Key considerations
Deep infiltrating endometriosis	High surgical complexity, frequent involvement of bowel, ureter, or bladder	Requires advanced imaging, disease mapping, and coordinated surgical expertise
Bowel or urinary tract involvement	Risk of silent organ dysfunction and irreversible damage	Multidisciplinary planning with colorectal and urologic surgeons is essential
Large or complex ovarian endometriomas	Increased risk of ovarian reserve impairment and surgical complications	Fertility preservation strategies and ovarian-sparing techniques should be considered
Refractory symptoms or prior surgery	Higher risk of complications and limited benefit from repeat surgery	Careful patient selection and realistic counseling are mandatory
Fertility preservation priority	Need to balance symptom control and reproductive outcomes	Integration with reproductive specialists improves decision-making

## Final considerations

Surgical management of endometriosis requires a selective, individualized, and patient-centered approach. Surgery should be regarded as one component of a comprehensive and longitudinal treatment strategy rather than a definitive solution. Decisions regarding indication, timing, extent of surgery, and referral level must integrate symptom burden, disease phenotype, impact on quality of life, and reproductive goals, supported by structured imaging and multidisciplinary evaluation when appropriate. Transparent counseling and shared decision making are essential to align surgical objectives with patient expectations and to optimize long-term outcomes.

## Data Availability

research data are available in the article.

## National Endometriosis Expert Committee of the Brazilian Federation of Gynecology and Obstetrics Associations (FEBRASGO)

President:

Ricardo de Almeida Quintairos

Vice-president:

Marcia Mendonça Carneiro

Secretary:

Carlos Augusto Pires Costa Lino

Members:

Beatriz Taliberti da Costa Porto
Carlos Alberto Petta
Helizabeth Salomão Abdalla Ayroza Ribeiro
João Nogueira Neto
João Sabino Lahorgue Da Cunha Filho
Julio Cesar Rosa e Silva
Marcia Cristina Franca Ferreira
Marco Aurelio Pinho De Oliveira
Marcos Tcherniakovsky
Mauricio Simoes Abrao
Omero Benedicto Poli Neto
Raquel Papandreus Dibi
Sergio Podgaec
Sidney Pearce Furtado

## Brazilian Society of Endometriosis

Paulo Ayroza Ribeiro

Vice President

Helizabet Salomão A. Ayroza Ribeiro

Scientific Director

Marcos Tcherniakovsky

Director of Communications

Luciano Gibran

Director of Education
